# The expression and clinical significance of miR-635 and miR-519d in gastric cancer patients

**DOI:** 10.3389/fgene.2026.1782820

**Published:** 2026-05-29

**Authors:** Jinjin Liu, Qiuping Gu, Cixiang Chen, Xiaohui Liu

**Affiliations:** 1 Department of Oncology, Ganzhou People’s Hospital, Ganzhou, Jiangxi, China; 2 Department of Gastroenterology, Ganzhou People’s Hospital, Ganzhou, Jiangxi, China; 3 The First Clinical Medical College, Gannan Medical University, Ganzhou, Jiangxi, China; 4 Department of Oncology, The First Affiliated Hospital of Gannan Medical University, Ganzhou, Jiangxi, China

**Keywords:** clinical pathological features, gastric cancer, microribonucleic acid-519d, microribonucleic acid-635, prognosis

## Abstract

**Objective:**

To investigate the expression and clinical significance of miR-635 and miR-519d in patients with gastric cancer.

**Methods:**

A total of 116 patients with gastric cancer admitted between July 2018 and July 2020 were enrolled. Gastric cancer tissues and matched adjacent tissues obtained during surgery were collected. The expression levels of miR-635 and miR-519d were detected by quantitative real-time PCR (qRT-PCR). Kaplan–Meier survival curves were used to analyze the relationship between miR-635 and miR-519d expression and patient prognosis. Cox regression analysis was performed to identify prognostic factors. ROC curves were plotted to assess the predictive value of miR-635 and miR-519d for prognosis, and the prognostic model was internally validated using the Bootstrap method.

**Results:**

The expression levels of miR-635 and miR-519d differed significantly between gastric cancer tissues and adjacent tissues (P < 0.05). miR-635 and miR-519d were associated with TNM stage, degree of differentiation, lymph node metastasis, and depth of infiltration (P < 0.05). Their expression levels in the death group were significantly lower than those in the survival group (P < 0.05). The AUC of the combined detection of miR-635 and miR-519d for predicting prognosis was 0.935, which was superior to that of either marker alone (Z combination vs miR-635 = 2.321, Z combination vs miR-519d = 2.043, P < 0.05). The 3-year survival rates of patients with moderately decreased miR-635 or miR-519d expression were higher than those of patients with severely decreased expression (χ^2^ = 26.038 and 14.255, respectively; P < 0.05). Multivariate Cox regression analysis showed that TNM stage, degree of differentiation, lymph node metastasis, and depth of infiltration were risk factors affecting prognosis (P < 0.05), whereas miR-635 and miR-519d were protective factors (P < 0.05). Internal validation showed a C-index of 0.935, and the Hosmer–Lemeshow test yielded χ^2^ = 7.786, P = 0.742.

**Conclusion:**

miR-635 and miR-519d are significantly downregulated in gastric cancer tissues and are associated with clinicopathological characteristics and prognosis. Their combined detection can effectively predict the prognosis of patients with gastric cancer.

## 1 Introduction

The clinical treatment of gastric cancer is radical surgical resection. However, gastric cancer has high invasiveness and early symptoms are not obvious, leading to a high incidence of metastasis (López et al., 2022). Often, it is discovered at a late stage, resulting in a high mortality rate and poor prognosis. Clinically, patients may experience discomfort in the upper abdomen and belching, which are easily overlooked, keeping the incidence rate high (Bray et al., 2018). The incidence of gastric cancer increases with age and is common in the elderly, but in recent years it has been increasingly affecting younger individuals, seriously threatening patient safety (Yang et al., 2023). Early gastric cancer lacks specific clinical symptoms, and some commonly used tumor markers have low specificity, leading to a still low 5-year survival rate for patients (Kim et al., 2021). Therefore, finding specific tumor markers is crucial for improving patient prognosis. MicroRNAs (miRNAs) can pair with target mRNAs to promote degradation of the target mRNA or inhibit protein translation. They are widely involved in regulating cell proliferation and apoptosis, acting as oncogenes or tumor suppressor genes in tumor development. miRNAs are abnormally expressed in malignant tumors and can serve as biomarkers for the prognosis of malignant tumorsAswathy, Chalos (Aswathy et al., 2024), (Salunkhe et al., 2025). Studies have found that miR-635 and miR-519d are involved in the progression of various malignant tumors and are abnormally decreased in non-small cell lung cancer (Tai et al., 2021; Wang et al., 2020). Currently, there is limited research on miR-635 and miR-519d in gastric cancer, so this study primarily explores the expression and clinical significance of miR-635 and miR-519d in gastric cancer patients.

## 2 Materials and methods

### 2.1 General information

**FIGURE 1:**
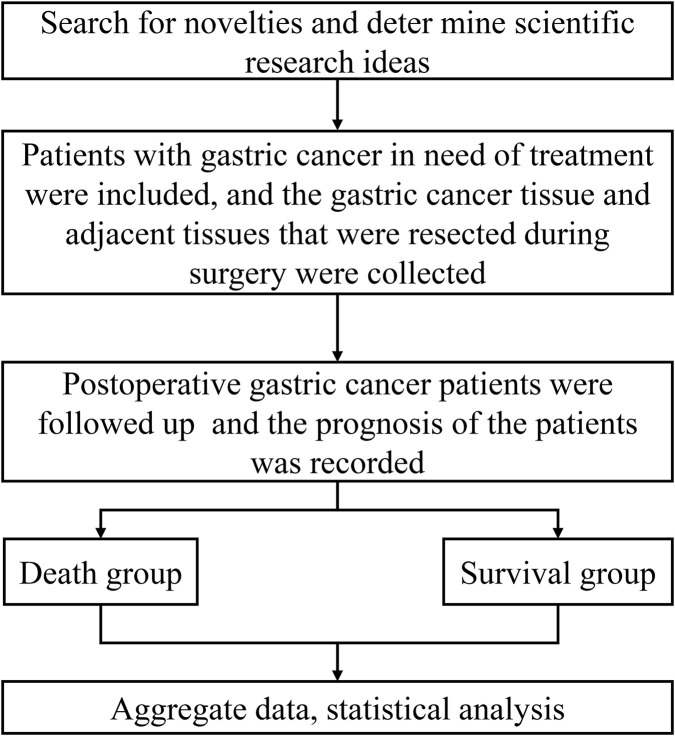
Flow chart of case collection.

A total of 116 gastric cancer patients were selected, treated in our hospital from July 2018 to July 2020. The study collected gastric cancer tissues and corresponding adjacent non-cancerous tissues (located ≥5 cm from the tumor margin and pathologically confirmed to contain no tumor cells)excised during surgery for radical resection for gastric cancer. Among these patients, 64 were male and 52 were female, aged between 35 and 81 years, with an average age of 60.50 ± 13.24 years. The case collection flow chart is shown in Figure 1. Inclusion criteria: (1) Postoperative pathological examination confirmed as gastric cancer; (2) All patients underwent radical surgical resection (Ajani et al., 2016); (3) Complete clinical and pathological data; (4) Patients signed consent forms. Exclusion criteria: (1) Preoperative radiotherapy or chemotherapy; (2) Concurrent other malignant tumors; (3) Significant organ failure; (4) Infectious diseases; (5) Immune and hematological diseases. This study was approved by the Ethics Committee of our hospital.

### 2.2 Methods

#### 2.2.1 Main reagents and Equipment

Trizol reagent, M-MLV reverse transcription kit (purchased from Beijing Kaishiyuan Company); NanoDrop ND-12000 spectrophotometer (purchased from Thermo Fisher Scientific, United States of America); Real-time quantitative PCR (qRT-PCR) instrument (purchased from Bio-Rad Laboratories, United States of America); SYBR Green Master Mix (2×) (purchased from Shanghai Beinuo Company).

#### 2.2.2 qRT-PCR for detection of miR-635 and miR-519d expression levels

All samples were rapidly placed into sterile cryovials within 30 min after surgical resection, immediately snap-frozen in liquid nitrogen, and then transferred to a −80 °C ultra-low-temperature freezer for long-term storage, with a storage period not exceeding 6 months. Total RNA from gastric cancer tissues and adjacent non-cancerous tissues was extracted using Trizol reagent. The concentration and purity of total RNA were assessed using a spectrophotometer. cDNA was synthesized using the M-MLV reverse transcription kit. Then, using cDNA as a template, the expression levels of miR-635 and miR-519d in the samples were detected by qRT-PCR, with U6 as the internal reference. Primer sequences are shown in Table 1. The qRT-PCR reaction volume was 20 µL. The expression of miR-635 and miR-519d was calculated using the 2^−ΔΔCt^ t method.

**TABLE 1:** qRT-PCR primer sequences.

Gene	Forward primers 5’--3′	Reverse primers 5’--3′
miR-635	AGG​ACG​GCT​CCT​CTA​ACC​AT	AGC​GGC​TCC​ACA​AGT​AAG​AC
miR-519d	AAC​CAG​CGC​ATG​GAC​AGT​TA	GAC​TTG​ACC​ACC​GAA​CCC​AT
U6	CTCGCTTCGGCAGCACA	AAC​GCT​TCA​CGA​ATT​TGC​GT

### 2.3 Follow-up

The enrolled patients were systematically followed up postoperatively through phone calls or hospital visits. The follow-up period started from the end of the surgery, lasting for 36 months, with the latest cutoff in July 2023. No patients were lost to follow-up, and the median follow-up time was 19 months. Patients were divided into survival and death groups.

### 2.4 Statistical analysis

Data was processed using SPSS 25.0 software. Quantitative data (conforming to normal distribution) were analyzed using the t-test and represented as ( $\bar{x}\pm s$ ). Categorical data were analyzed using the χ2 test, represented by n. The relationship between miR-635 and miR-519d and the prognosis of gastric cancer patients was analyzed using the Kaplan-Meier survival curve. Factors affecting the prognosis of gastric cancer patients were analyzed using COX regression. ROC curves were drawn to analyze the predictive value of miR-635 and miR-519d for the prognosis of gastric cancer patients. AUC comparisons were performed using the DeLong test, and the prognostic prediction model for patients with gastric cancer was validated using the Bootstrap method. A P-value <0.05 was considered statistically significant.

## 3 Results

### 3.1 Comparison of miR-635 and miR-519d expression levels and their relationship with clinicopathological characteristics

As shown in Table 2, there was a significant difference in the expression levels of miR-635 and miR-519d between gastric cancer tissues and adjacent non-cancerous tissues (P < 0.05). As shown in Table 3, based on the mean levels of miR-635 (0.45) and miR-519d (0.51), the patients were grouped. miR-635 < 0.45 was classified as the severely decreased group, and ≥ 0.45 as the moderately decreased group; miR-519d < 0.51 as the severely decreased group, and ≥ 0.51 as the moderately decreased group. The expression of miR-635 and miR-519d was related to clinicopathological characteristics, including TNM stage, degree of differentiation, lymph node metastasis, and invasion depth (P < 0.05). They were not related to other factors (P > 0.05).

**TABLE 2:** Comparison of miR-635 and miR-519d expression levels in gastric cancer tissues and paracancerous tissues.

Tissue	n	miR-635	miR-519d
Paracancerous tissue	116	1.02 ± 0.23	1.01 ± 0.22
Gastric cancer tissue	116	0.45 ± 0.12	0.51 ± 0.14
*t*	-	23.664	20.651
*P*	-	<0.001	<0.001

**TABLE 3:** Relationship between the expression levels of miR-635 and miR-519d and clinicopathological features in gastric cancer tissues[n(%)].

Pathologic features	n(116)	miR-635		x^2^	*P*	miR-519d		x^2^	*P*
		Moderately decreased n = 57(%)	Severely decreasedn = 59(%)			Moderately decreased n = 55(%)	Severely decreasedn = 61(%)		
Age (years)	​	​	​	0.346	0.556	​	​	0.411	0.522
>60	50	23(46.00)	27(54.00)	​	​	22(44.00)	28(56.00)	​	​
≤60	66	34(51.52)	32(48.48)	​	​	33(50.00)	33(50.00)	​	​
Gender	​	​	​	0.836	0.361	​	​	1.564	0.211
man	64	29(45.31)	35(54.69)	​	​	27(42.19)	37(57.81)	​	​
Woman	52	28(53.85)	24(46.15)	​	​	28(53.85)	24(46.15)	​	​
TNM staging	​	​	​	8.184	0.004	​	​	4.727	0.030
I ∼ II stage	68	41(60.29)	27(39.71)	​	​	38(55.88)	30(44.12)	​	​
III ∼ IVstage	48	16(33.33)	32(66.67)			17(35.42)	31(64.58)		
Tumor diameter	​	​	​	0.318	0.573	​	​	0.333	0.564
<4.0 cm	56	26(46.43)	30(53.57)	​	​	25(44.64)	31(55.36)	​	​
≥4.0 cm	60	31(51.67)	29(48.33)			30(50.00)	30(50.00)		
Degree of differentiation	​	​	​	7.204	0.007	​	​	7.613	0.006
Low differentiation	47	16(34.04)	31(65.96)	​	​	15(31.91)	32(68.09)	​	​
Medium and high differentiation	69	41(59.42)	28(40.58)			40(57.97)	29(42.03)		
Lymph node metastases	​	​	​	10.669	0.001	​	​	6.701	0.010
Yes	46	14(30.43)	32(69.57)	​	​	15(32.61)	31(67.39)	​	​
No	70	43(61.43)	27(38.57)	​	​	40(57.14)	30(42.86)	​	​
Depth of infiltration	​	​	​	9.560	0.002	​	​	4.146	0.042
T1∼T2	71	43(60.56)	28(39.44)	​	​	39(54.93)	32(45.07)	​	​
T3∼T4	45	14(31.11)	31(68.89)			16(35.56)	29(64.44)		

### 3.2 Comparison of miR-635 and miR-519d expression levels between the survival and death groups and their predictive value for prognosis

**FIGURE 2:**
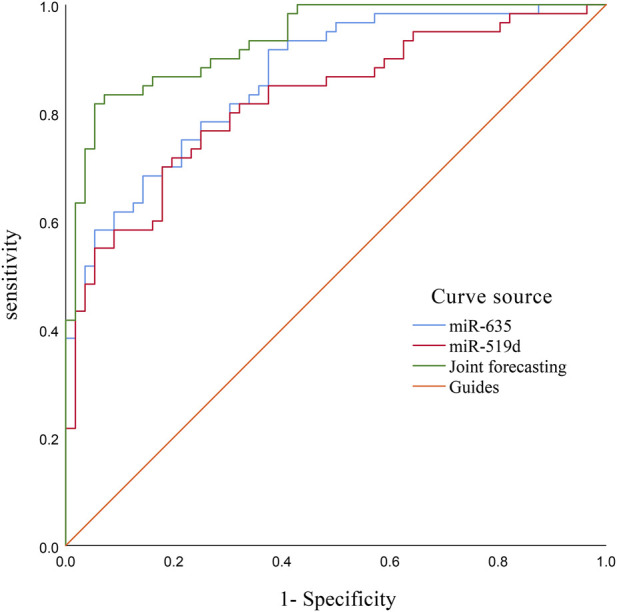
The predictive value of miR-635 and miR-519d expression levels in the prognosis of gastric cancer patients.

As shown in Table 4, the expression levels of miR-635 and miR-519d were significantly lower in the death group compared to the survival group (P < 0.05).According to the ROC curve, the AUC for miR-635 in predicting the prognosis of gastric cancer patients was 0.864, for miR-519d it was 0.822, and for their combined prediction, it was 0.935. The combined prediction was superior to individual predictions (Z _combined vs miR_-635 = 2.321, Z _combined vs miR_-519d = 2.043, P < 0.05 each), as shown in Figure 2 and Table 5.

**TABLE 4:** Comparison of miR-635 and miR-519d expression levels between the surviving and deceased groups.

Group	n	miR-635	miR-519 d
Survival group	60	0.64 ± 0.15	0.69 ± 0.17
Death group	56	0.25 ± 0.08	0.31 ± 0.10
*t*	-	17.293	14.541
*P*	-	<0.001	<0.001

**TABLE 5:** The value of miR-635 and miR-519d expression levels in predicting the prognosis of gastric cancer patients.

Project	AUC	95%CI	Sensitivity(%)	Specificity(%)	Truncated value
miR-635	0.864	0.801∼0.928	69.54	84.12	0.312
miR-519d	0.822	0.746∼0.897	75.37	78.62	0.341
Joint forecasting	0.935	0.893∼0.976	84.54	76.31	-

### 3.3 Relationship between the expression of miR-635 and miR-519d in gastric cancer tissues and prognosis

**FIGURE 3:**
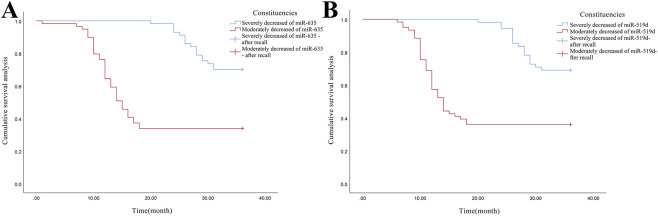
Relationship between the Expression of miR-635 and miR-519d in Gastric Cancer Tissues and Prognosis. **(A)** Relationship between miR-635 and prognosis; **(B)** Relationship between miR-519d and prognosis.

The 3-year survival rate of patients with moderately decreased miR-635 expression (40/57, 70.18%) was higher than that of patients with severely decreased (20/59, 33.90%) (*χ* ^2^ = 26.038, P < 0.05), as shown in Figure 3A. The 3-year survival rate of patients with moderately decreased miR-519d expression (38/55, 69.09%) was higher than that of patients with severely decreased (22/61, 36.07%) (*χ* ^2^ = 14.255, P < 0.05), as shown in Figure 3B.

### 3.4 Analysis of factors influencing the prognosis of gastric cancer patients

Using whether gastric cancer patients died within 3 years as the dependent variable (yes = 1, no = 0), and variables such as miR-635, miR-519d, TNM stage, degree of differentiation, lymph node metastasis, and invasion depth as independent variables, a COX regression analysis was conducted. The assignment of each variable is shown in Table 6. As indicated in Table 7, univariate COX regression analysis showed that clinical pathological characteristics are risk factors affecting the prognosis of gastric cancer patients (P < 0.05), while miR-635 and miR-519d are protective factors (P < 0.05). Multivariate COX regression analysis also indicated that clinical pathological features are risk factors (P < 0.05), and miR-635 and miR-519d are protective factors (P < 0.05).

**TABLE 6:** Assignment methods for each variable.

Variable	Assignment method
miR-635	Moderately decreased = 0, severely decreased = 1
miR-519d	Moderately decreased = 0, severely decreased = 1
TNM staging	I ∼ IIstage = 0,III ∼ IVstage = 1
Degree of differentiation	Medium and high differentiation = 0, low differentiation = 1
Lymph node metastases	No = 0,Yes = 1
Depth of infiltration	T1∼T2 = 0,T3∼T4 = 1

**TABLE 7:** COX regression analysis of the influencing factors of prognosis in gastric cancer patients.

Factor	Univariate analysis			Multivariate analysis		
	OR	95%CI	*P*	OR	95%CI	*P*
miR-635	0.578	0.426–0.785	<0.001	0.501	0.369–0.680	<0.001
miR-519d	0.418	0.308–0.568	<0.001	0.467	0.344–0.634	<0.001
TNM staging	3.278	2.576–4.172	0.000	2.147	1.151∼4.004	0.016
Degree of differentiation	5.124	2.210–11.879	0.000	1.771	1.201–2.611	0.004
Lymph node metastases	4.012	3.153–5.106	0.000	3.454	1.353–8.815	0.010
Depth of infiltration	2.468	1.321–4.612	0.004	3.547	1.554∼8.095	0.002

### 3.5 Internal validation of the prognostic prediction model for patients with gastric cancer

**FIGURE 4:**
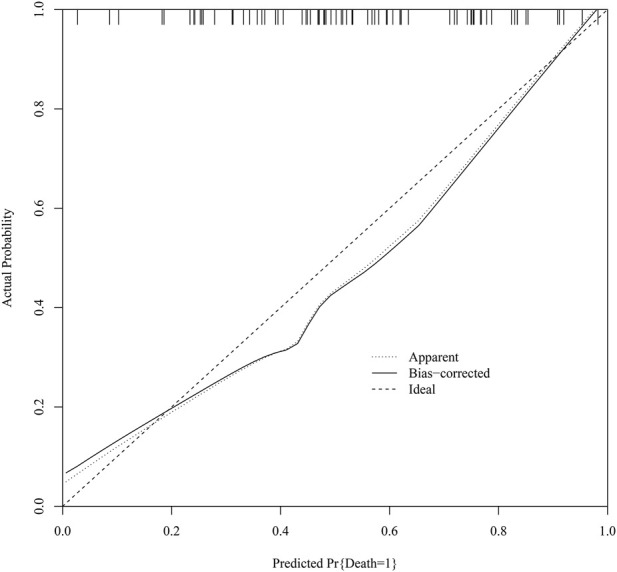
Internal validation of the prognostic prediction model for gastric cancer patients.

Internal validation using the Bootstrap method (1,000 repetitions) showed that the model had a C-index of 0.935 for predicting the prognosis of patients with gastric cancer. The Hosmer–Lemeshow test yielded χ² = 7.786 and P = 0.742, indicating good agreement (Figure 4).

## 4 Discussion

The mechanism of gastric cancer involves multiple stages and is one of the most common malignant tumors in China. Significant progress has been made in the treatment of gastric cancer in China, yet the survival rate remains low in most regions (Norwood et al., 2022). Clinical symptoms of gastric cancer patients include nausea, anorexia, peptic ulcers, etc., posing a serious threat to the patients' health and life (Chen et al., 2025). With the ongoing development of molecular biology, some therapeutic strategies targeting gastric cancer have been clinically developed (Hou et al., 2023). However, the prognosis for gastric cancer patients remains poor. Therefore, finding molecular biomarkers related to the prognosis of gastric cancer is crucial for clinical diagnosis, treatment, and prognosis assessment.

miRNAs, located in eukaryotes, regulate target genes post-transcriptionally and participate in various biological processes, affecting the proliferation and migration of malignant tumor cells (Ma et al., 2020a; Wu et al., 2024). Dysregulation of miRNAs is related to the progression of gastric cancer, and recent studies have found that various miRNAs are involved in the development of gastric cancer (Yu et al., 2024). miR-635 is underexpressed in gastric cancer tissues and may also negatively regulate kinesin family member C1 (KIFC1). miR-635 can target KIFC1 to inhibit the proliferation, migration,and invasion of gastric cancer cells,and can also suppress the proliferation and invasion of gastric cancer cells by regulating KIFC1(16). Asiaticoside can inhibit the proliferation and migration of gastric cancer by increasing the expression of miR-635, thereby inducing endoplasmic reticulum stress, becoming a potential target for gastric cancer treatment (Zhang et al., 2022). miR-635 is downregulated in non-small cell lung cancer tissues. Circ_0102231 interacts with miR-635 and regulates the miR-635/NOVA2 axis, thereby inactivating the PI3K/AKT signaling pathway and promoting the progression of non-small cell lung cancer (Liu et al., 2023). The results of this study showed that miR-635 expression was decreased in gastric cancer, which is consistent with previous studies, and was associated with the clinicopathological characteristics of gastric cancer, including TNM stage, degree of differentiation, lymph node metastasis, and depth of invasion, suggesting that the downregulation of miR-635 may contribute to gastric cancer progression through loss of its tumor-suppressive effect. Previous studies have shown that miR-635 can directly target and inhibit KIFC1, thereby suppressing the proliferation, migration, and invasion of gastric cancer cells. Therefore, reduced miR-635 expression may weaken this inhibitory effect on KIFC1 and consequently facilitate tumor progression (Cao et al., 2020).

miR-519d also plays an important role in the development of various cancers, acting either as a tumor suppressor or an oncogene. miR-519d is underexpressed in breast cancer tissues and can target Ki-67 to exert an anti-cancer effect in breast cancer (Ma et al., 2020b). miR-519d is decreased in thyroid cancer tissues, closely related to poor prognosis, and can promote the progression of thyroid cancer by regulating FOXQ1(20). miR-519d is significantly reduced in oral squamous cell carcinoma tissues, and its reduction is also related to lymph node metastasis, tumor staging (advanced), and overall survival (poor). miR-519d is an epithelial-mesenchymal transition regulator in oral squamous cell carcinoma cells, and reduced miR-519d expression promotes invasion and metastasis by targeting matrix metalloproteinase 3 (Jin et al., 2019). miR-519d is also significantly reduced in gastric cancer cells, and long non-coding RNA HCP5 promotes gastric cancer cell proliferation and cisplatin resistance by regulating miR-519d (Zhang and Wang, 2021). Our study results show that the expression levels of miR-519d was decreased in gastric cancer tissues, similar to previous studies, and miR-519d is related to the clinicopathological characteristics of gastric cancer,including TNM stage, degree of differentiation, lymph node metastasis, and invasion depth, suggesting that reduced miR-519d expression may contribute to gastric cancer progression through attenuation of its tumor-suppressive function. FOXQ1 is considered an oncogenic transcription factor that promotes tumor cell proliferation, invasion, and metastasis. Previous studies have shown that miR-519d can negatively regulate FOXQ1. Therefore, when miR-519d expression is reduced, its inhibitory effect on FOXQ1 is weakened, which may in turn promote the proliferation and invasion of gastric cancer cells (Li et al., 2021). Further studies found that during follow-up for prognosis, the expression levels of miR-635 and miR-519d in the death group were significantly lower than in the survival group. Additionally, gastric cancer patients with moderately decreased expression of miR-635 and miR-519d had higher 3-year survival rates than those with severely decreased, indicating their relevance to the prognosis of gastric cancer patients. COX regression analysis showed that clinical pathological characteristics are risk factors for the prognosis of gastric cancer patients, while miR-635 and miR-519d are protective factors, indicating that miR-635 and miR-519d are closely related to patient prognosis and can promote the progression of gastric cancer. According to the ROC curve, the combined prediction of miR-635 and miR-519d for the prognosis of gastric cancer patients is superior to their individual predictions, suggesting that their combination can more effectively predict the prognosis of gastric cancer patients, thereby enabling prioritization of relevant treatments and improving patient outcomes, helping clinicians assess patients’ prognostic risk, guide treatment decisions, and facilitate the adoption of more aggressive postoperative adjuvant therapy or closer follow-up strategies.

In summary, the expression levels of miR-635 and miR-519d are significantly reduced in gastric cancer tissues. Both are related to the clinical pathological characteristics and prognosis of gastric cancer patients, and their combined use can effectively predict the prognosis of gastric cancer patients. This study has several limitations. It was a single-center study with a relatively small sample size, and no external validation was performed, which may limit the generalizability of the results. In addition, the follow-up period was relatively short, which may have affected the accuracy of the results. Functional experiments to verify the roles of these miRNAs were also lacking. Moreover, all patients were from the same geographic region. In future studies, the sample size should be expanded and external validation should be performed to further provide precise molecular targets for the diagnosis and treatment of gastric cancer.

## Data Availability

The original contributions presented in the study are included in the article/supplementary material, further inquiries can be directed to the corresponding author.
